# Evaluation of parents’ views about etiologic factors of severe early childhood caries: A qualitative study

**DOI:** 10.15171/joddd.2019.007

**Published:** 2019-04-24

**Authors:** Arian Hesam Arefi, Hoda Shamsaddin, Mojgan Balvardi, Hamidreza Poureslami, Masumeh Danesh, Mahsa Sayadizadeh

**Affiliations:** ^1^Department of Orthodontics, Faculty of Dentistry, Tehran University of Medical Sciences, Tehran, Iran; ^2^Department of Oral & Maxillofacial Surgery, Faculty of Dentistry, Zahedan University of Medical Sciences, Zahedan, Iran; ^3^Kerman Social Determinants on Oral Health Research Center & Oral and Dental Diseases Research Center & Department of Pediatric Dentistry, Faculty of Dentistry, Kerman University of Medical Science, Kerman, Iran; ^4^Department of Pediatric Dentistry, Faculty of Dentistry, Kerman University of Medical Science, Kerman, Iran

**Keywords:** Breast milk lactose, early childhood carries, maternal stress, mode of delivery, qualitative study

## Abstract

***Background***. Severe early childhood caries (S-ECC) is a rapid form of dental caries that firstly affects primary upper incisors of children less <3 years of age and can cause interference in child’s health by pain, nutritional deficiencies and sleep disorders. It seems there are many unknown factors in the etiology as well as progression of S-ECC. The aim of this study was to assess parents' views in this relation.

***Methods***. In this qualitative study parent’s views and their 1‒3-year-old children were studied when they visited pediatric medical clinics in Kerman. After cleaning the children teeth and examination of them to discover caries, they were placed in 2 groups with or without S-ECC. Then each parent was interviewed separately and their comments were collected and studied. Examinations and interviews continued with parents until they did not express anything new.

***Results***. Parents of children without S-ECC had better understanding about S-ECC related factors than parents of children with S-ECC and greater number of them (without significant differences) expressed known reasons for occurrence of S-ECC. There were differences among known reasons and proposed reasons mostly in parents of children with S-ECC, although the differences were not significant. Maternal stress and amount of breast milk’s lactose were factors that were reported by some parents while there were unknown factors related to the etiology of S-ECC. Furthermore, no parents mentioned factors like saliva or mode of delivery.

***Conclusion***. The most important achievement of the study was the attention of some parents to the role of their chronic stress in the occurrence of S-ECC. Another important consideration was that none of the parents mentioned the important role of the quality and quantity of saliva in preventing S-ECC, which should be promoted in the community

## Introduction

Dental caries is one of the most common chronic diseases in childhood and its prevalence has increased in preschool children in recent decades. Currently, many children do not enjoy proper general and dental health due to uncontrolled and active dental caries. Although the available data confirm that much has been learned about prevention of dental caries in recent decades, the multi-factorial nature of dental caries prevents a decrease in the unmet treatment needs, especially in children, despite knowledge about the effective techniques to prevent dental caries.^1^

The prevalence rate of early childhood caries (ECC) as an infectious transmissible disease in preschool children is different in various communities. It has been reported that ECC is one of the most common chronic diseases of childhood in the United States. Its prevalence is 5 times that of asthma in children.^2^

In Iran, the prevalence of ECC has been reported to be different in different parts of the country. Its prevalence has been reported 17.3%^3^ and 21.1%^4^ in Tehran. However, its prevalence in Qazvin has been reported to be 9.9%^5^ and 19.5%,^6^ with 19%^7^ in Mashhad. In Kerman, the prevalence of ECC was evaluated three times in 2000, 2003 and 2005, which yielded prevalence rates of 44%, 39% and 45.1%, respectively,^8-10^ which is unfortunately very high compared to that in other cities and countries, necessitating further studies on this condition and on how to prevent and manage it.

Different age groups have been used for evaluation of prevalence of ECC. Various studies have established a strong relationship between frequent use of food products containing sucrose, lactose, fructose and ECC.^11-13^

Poureslami et al^14^ evaluated the relationship between ECC and 3‒5-year-old children’s height, weight and head circumference in kindergartens in Rafsanjan, Iran and concluded that there was no significant relationship between ECC and the variables above.

The aim of the present qualitative study was to determine the opinions of affected children’s parents about reasons for the occurrence of S-ECC.

Since various unknown factors might be responsible for the occurrence of S-ECC, an attempt was made to determine new possible etiologic factor(s) based on the opinions of parents so that measures can be adopted to manage these factors to control this condition. On the other hand, it would be possible to educate the parents in case of incorrect opinions among them. In this context, it would be possible to make the parents familiar with possible and specific reasons for S-ECC so that it would be prevented in future in other children of the family and also prevent its progression in the affected children.

## Methods

The present qualitative study was carried out on 1‒3-year-old children in Kerman, Iran. All the generally healthy children (without physical and/or mental disabilities, as well as medical conditions) <3 years of age, who referred to clinics of pediatricians in Kerman for routine checkups or for simple therapeutic needs such as common cold, were included in the study. Two groups of children were evaluated: children with S-ECC (the case group) and children without S-ECC (the control group).

The inclusion criteria consisted of the following: general health, an age range of 12‒36 months, proper physical and mental growth, and minimum cooperation for examination of teeth.

The dental examinations of the subjects were carried out under a 100-W lamp, using a dental explorer and a disposable dental mirror. First, the four upper anterior teeth were cleaned with a piece of sterile gauze and the presence or absence of caries on these teeth (S-ECC: remarkable enamel decalcification and/or cavitation) was recorded. Then the parents were interviewed about their opinion in relation to the possible reasons for dental caries or its absence in their children. These short interviews were recorded on a tape. Examinations and interviews continued with 82 parents until they did not express anything new. Therefore, was decided to stop interviews with other parents.

For ethical reasons, the parents were justified about the aims of the study and informed consent forms were obtained.

Interviews recorded on the tape were evaluated and the required data were extracted and recorded on questionnaires. After description and classification of data, the data collected from the questionnaires were analyzed. Due to the qualitative nature of the study, it was not possible to use a specific statistical test for the analysis of data.

## Results

The mean (SD) of the age of children in this study was 2.76(0.94) year. Other demographic variables as well as the parents’ opinions are presented in Tables 1 and 2.

**Table 1:** Descriptive statistics of some demographic variables

**Variables**		**Frequency (%)**
**Gender of the children**	Female	39 (47.56)
	Male	43 (52.44)
**Educational level of the parents**	High school and lower	32 (39.02)
	Bachelor's degree	42 (51.22)
	Master's degree	8 (9.76)
**Groups**	Without caries	16 (19.51)
	With caries	66 (80.49)

**Table 2:** Descriptive statistics of the parents’ opinions

**Variables based on the parents' opinions**		**Groups**	
		**Without caries**	**With Caries**
**Iron drops cause dental caries.**	Yes	0	28 (42.42%)
	No	16 (100%)	38 (57.58%)
**Dental caries is a hereditary condition.**	Yes	2 (12.5%)	9 (13.64%)
	No	14 (87.5%)	57 (86.36%)
**Not brushing the teeth causes dental caries.**	Yes	11 (68.75%)	15 (22.73%)
	No	5 (31.25%)	51 (68.29%)
**Intake of sweet snacks causes dental caries.**	Yes	11 (68.75%)	29 (43.94%)
	No	5 (31.25%)	37 (56.06%)
**Calcium deficiency causes dental caries.**	Yes	1 (6.25%)	5 (7.58%)
	No	15 (93.75%)	61 (92.42%)
**Mother's diabetes causes dental caries.**	Yes	0	3 (4.55%)
	No	16 (100%)	63 (95.45%)
**Drinking milk/breastfeeding during sleep cases dental caries in children.**	Yes	7(43.75%)	24 (36.36%)
	No	9 (56.25%)	42 (63.64%)
**Tooth structure deficiency causes dental caries.**	Yes	2 (12.5%)	2 (3.03%)
	No	14 (87.5%)	64 (96.97%)
**Early eruption of premature tooth causes dental caries.**	Yes	0	2 (3.03%)
	No	16 (100%)	64 (96.97%)
**Use of a pacifier causes dental caries.**	Yes	1 (6.25%)	5 (7.58%)
	No	15 (93.75%)	61 (92.42%)
**Systemic conditions cause dental caries.**	Yes	0	3 (4.55%)
	No	16 (100%)	63 (95.45%)
**Formulated milk causes dental caries.**	Yes	1 (6.25%)	4 (6.06%)
	No	15 (93.75%)	62 (93.94%)
**Mouth breathing causes dental caries.**	Yes	0	2 (3.03%)
	No	16 (100%)	64 (96.97%)
**Mother’s stresses during breastfeeding period cause dental caries.**	Yes	0	5 (7.58%)
	No	16 (100%)	61 (92.42%)
**High lactose levels in some mothers’ milk cause dental caries.**	Yes	0	5 (7.58%)
	No	16 (100%)	61 (92.42%)
**Low quality of foodstuff causes dental caries.**	Yes	0	3 (4.55%)
	No	16 (100%)	63 (95.45%)
**Transfer of microbes from the oral cavity of the mother causes dental caries.**	Yes	1 (6.25%)	1 (1.52%)
	No	15 (93.75%)	65 (98.48%)

## Discussion

The chief aim of the present study was to evaluate etiologic factors for S-ECC based on the parents’ opinions in order to acquire deeper knowledge about this condition. The principal data were collected through interviews with the parents of affected and unaffected children.

If ECC is not diagnosed and managed early, widespread dental problems, medical complications, social problems and changes in the quality of life of the affected children will ensue.^15^

The list of variables that either directly or indirectly affect dental caries in children is very long. The list consists of clinical/biological factors (such as previous caries experience in the child and his/her caregiver, quality and quantity of microbial plaque, gingivitis, saliva, tooth developmental anomalies, medical conditions, mode of delivery, igA levels of the child’s saliva, and genetic and local factors (such as exposure to fluoride, use of antibiotics) and behavioral/ demographic factors (such as diet, oral hygiene habits, age, parents’ attitudes, the child’s mood, beliefs, the educational level of the caregiver, socioeconomic status, status of the supportive insurance and access to dental care).^16,17^

In the present qualitative study, the parents reported 17 items during interviews in relation to their opinions about factors responsible for or effective in the occurrence of S-ECC. These interviews continued until the parents did not mention any items other than these 17 items:

### Intake of Sweet Snacks

Intake of sweet snacks was reported as the etiologic factor for S-ECC by the majority of parents (43.94%) whose children were affected by the condition and by 68.75% of those whose children were not affected. Frequent intake of snakes containing carbohydrates leads to the production of acid in the dental plaque, resulting in the destruction of enamel.^15^ Fortunately, this opinion of the parents shows that they have proper awareness about one of the main etiologic factors for dental caries; this is very important by considering the fact that almost 70% of parents whose children were not affected reported it, i.e. they prevented dental caries in their children through their knowledge about this important fact.

### Iron Drops as Etiologic Agents for Dental Caries

A total of 42% of parents whose children had S-ECC reported iron drops as an etiologic agent for the condition; however, none of the parent of caries-free children believed that iron drops were responsible for dental caries. Many dentists meet parents during their career, who believe iron drops are etiologic agents for dental caries; however, no studies to date have shown that use of iron drops is an etiologic factor for dental caries.^18,19^ In fact, the idea that iron drop is an etiologic factor for dental caries originates from the concurrent occurrence of S-ECC and the period during which iron drops are administrated; therefore, iron pigments easily stain pitted or carious enamel. This does not easily take place on intact enamel with a smooth surface. An in vitro study showed that iron drops even have antibacterial effects and might exert anticariogenic effects.^20^ A study showed that a combination of iron drops and multi-vitamin drops can erode the enamel surface of deciduous teeth; therefore, they might increase the susceptibility of teeth to ECC.^21^

### Breastfeeding and Drinking Milk During Sleep

A total of 36% of parents whose children had S-ECC and 43% of parents whose children did not have S-ECC believed that feeding the child with milk during sleep causes ECC. Many studies have confirmed this fact.^22,23^

### Not Brushing the Teeth Results in Dental Caries

This was the opinion of approximately 23% of parents whose children had S-ECC and approximately 69% of parents whose children were caries-free. Not brushing the teeth results in the accumulation of bacteria and their early colonization on the surfaces of deciduous teeth and the occurrence of dental caries has been established in many studies.^24^ Fortunately, a large proportion of parents had such an opinion. Therefore, attempts should be made to improve the performance of such parents by improving the quality and quantity of tooth brushing in children, which will decrease the rate of dental caries.

### Dental Caries Is a Hereditary Condition

This was the opinion of approximately 14% and 12% of parents who had children with and without S-ECC, respectively. Studies have shown that although genetic factors and heredity affect susceptibility to caries, environmental factors such as oral hygiene and proper diet affect the role of heredity in dental caries, i.e. children who are susceptible to dental caries due to genetic factors and heredity will not be affected by dental caries if they have good oral hygiene and a proper diet. In contrast, children whose parents have healthy teeth will be affected by dental caries if they have poor oral hygiene and diet.^25^ Therefore, it is necessary to remind parents with such opinions that environmental factors can overcome hereditary factors and they should not forget intervention, believing that dental caries in their family is a hereditary problem, and they should not forget their children’s diet and ignore teaching oral hygiene measures to their children.

### Calcium Deficiency and Dental Caries

Less than 10% of parents in both groups believed that calcium deficiency was an etiologic factor for S-ECC. Studies have shown that calcium deficiency in the enamel of deciduous teeth compromises the tooth structure, increasing susceptibility to dental caries. Such calcium deficiency might be due to the mother’s diet during pregnancy and the time of calcification of the enamel of deciduous teeth, premature birth and low birth weight.^26^

### Mother's Diabetes and Dental Caries

Approximately 5% of parents believed that the mother’s diabetes was an etiologic factor for caries in deciduous teeth. No study is available to show that diabetic mothers have children with a higher rate of dental caries, and a high glucose level in the mother’s serum, either during pregnancy or after child birth, cannot affect the susceptibility of teeth to caries or aggravate caries.

### Tooth Structure Deficiency

Some parents believed that tooth structure deficiencies are effective in dental caries. ‘Tooth structure anomalies’ in their opinion meant a deficiency in the mineral content of teeth such as calcium and phosphate or the thickness of tooth enamel (quality and quantity of enamel). As discussed above studies have shown that disturbances and deficiencies in calcification of enamel in deciduous teeth during pregnancy result in the development of enamel with poor quality and quantity.^27^

A study showed that lack of weight gain of the fetus during 25‒28 weeks of gestational age, which is a period for rapid growth and is concomitant with development of the enamel of maxillary deciduous incisors, might result in poor enamel in such teeth, making them more susceptible to ECC.^28^

### Early and Premature Eruption of Teeth

Less than 5% of parents believed that early eruption of premature teeth results in S-ECC. This opinion has no scientific basis and no study has supported it. Although early eruption of teeth exposes them to other etiologic agents earlier, when the child has good oral hygiene and a proper diet, this early eruption will not have a significant effect on the occurrence of caries.^29^

### Use of a Pacifier

More than 5% of the parents believed that use of a pacifier results in dental caries. Studies have shown that use of a pacifier can induce dental caries under some conditions, including repeated covering of the pacifier with food items and sweet liquids such as honey and jam, followed by placing it in the child’s mouth to pacify him/her, or placing the pacifier in the oral cavity by the mother or the caregiver, resulting in its contamination with their saliva, which is laden with *S. mutans*; this way the bacterial species are transferred into the child’s oral cavity, increasing the risk of dental caries (vertical transmission of bacteria).^30^

### Systemic Conditions

Approximately 5% of the parent believed that systemic problems lead to S-ECC. Such an idea has been evaluated, and it has been confirmed that systemic conditions can contribute to S-ECC through three indirect ways:

1) When the mother is pregnant, if she is affected by certain systemic conditions, the normal growth and development of teeth in the fetus (formation of enamel matrix or calcification of enamel) is affected and teeth will develop with poor enamel.
2) After birth, a systemic condition in the child will direct the parent’s attention to the condition and the parents might ignore the child’s oral health.
3) Medications that a child takes for the treatment of his/her systemic condition might contain sweeteners that induce dental caries or aggravate them, such as syrups containing sweeteners.^31^

### Formulated Milk

A total of 6% of parents believed that formulated milk induces dental caries. Studies have shown that formulated milk has a higher potential to induce S-ECC compared to human and bovine milk. A study showed that if the cariogenic potential of sucrose is designated a value of ‘1’, this potential in relation to formulated milk ranges from 0.36 to 0.10, depending on the brand, and milk formulations poor in iron content exhibit great cariogenic effects compared to formulations rich in iron and soya-derived formulations. The cariogenic potential of bovine milk was only 0.05. Based on the results of the same study, milk formulations containing sucrose or corn syrup were more cariogenic than lactose-containing milk formulations.^32^

Another study showed that if the cariogenic potential of sucrose is designated a value of ‘1’, this value is 1.26 for Coca Cola, 0.88 for honey, 0.2 for human milk and 0.01 for bovine milk.^33^

### Mouth Breathing

Approximately 3% of the parents attributed their children’s dental caries to mouth breathing. No study is available to indicate that mouth breathing in very young children will contribute to the occurrence of S- ECC.

### Mother’s Stresses during Pregnancy orBbreastfeeding

Approximately 8% of mothers believed that stresses during pregnancy and breastfeeding were responsible for S-ECC in their children. Since the ancient times, mothers in Kerman believe that giving ‘boil mother milk’ to children is responsible for the majority of problems in children, i.e. if the mother is affected by stress and anxiety during breastfeeding period and has psychological problems, the quality and quantity of her milk will be affected and such milk, with an unfavorable quality, will affect the child’s body health. In the present study, some mothers laid great emphasis on this and believed that their emotional status affected their milk, which in turn affected their children. Recent studies have shown that the mother’s chronic stress will increase the rate of dental caries in the child.^34^

### A High Level of Lactose in Mother's Milk

Less than 10% of parents believed that a high level of lactose in human milk causes S-ECC. As discussed previously, milk formulations containing sucrose are more cariogenic than those containing lactose and human milk with 7% of lactose, which in turn is more cariogenic than bovine milk with 4% of lactose.^35^ In this study, some parents believed that the milk of some mothers contains a higher amount of lactose and therefore such milk has a greater role in S-ECC. No study is available to show the lactose concentration rates of the milk of mothers who have children with S-ECC. However, a study in Kerman showed no significant differences in the salivary glucose concentrations between children with ECC and those without ECC.^36^

### Low Quality of Foodstuffs Consumed by the Children

Approximately 5% of mothers believed that the low quality of foodstuffs consumed by children causes S-ECC. Undoubtedly, if no attention is paid to the optimal concentration of calcium in pasteurized milk consumed by children during its processing in the factory and its packaging or if extra calcium is not added to it, or if cocoa is added to it, sufficient calcium will not be available to strength the enamel of deciduous teeth. In addition, if there is insufficient fluoride in the drinking water or food consumed by the child, there will be a higher rate of S-ECC. Studies have shown that the prevalence of ECC is >40% in Kerman,^8^ while an unpublished report indicates that its prevalence in Koohbanan is much lower than that in Kerman, which might be attributed to the higher fluoride content of drinking water and foodstuffs produced in Koohbanan compared to Kerman.^37^

### Transfer of Microorganisms from the Parents’ Oral Cavities

A small number of parents believed that transfer of microbes from the parents’ oral cavities was responsible for S-ECC. Several studies have proved this transfer, which has been defined as vertical transfer from the mother and as horizontal transfer from the oral cavities of other relatives and family members.^38^ This transfer has a key role in the acquisition of *S. mutans* by the child and colonization of his/her oral cavity, leading to dental caries; since a small number of parents were aware of such an important issue, it is necessary to inform the mothers of this issue in different ways.

Unfortunately, none of the parents mentioned the important role of the quality and quantity of the child’s saliva in the occurrence of S-ECC; however, saliva is laden with many defense factors and has an important role in protection against S-ECC.

In addition, none of the mothers mentioned the possible role of mode of delivery (natural birth or caesarian). Studies on the subject have yielded contradictory results. A study in 2005 showed a higher rate of ECC in children born through caesarian section.^38^ Another study in Iran has confirmed this.^39^ However, a more recent study in 2013 refuted this and reported a higher rate of ECC in children born naturally.^40^

Limitation of the study was reluctance of some parents to record their voice, which decreased by continuation of the interview.

## Conclusion

In the present qualitative study, the parents reported 17 items as etiologic factors for ECC with different percentages. Reporting some of these factors is a source of delight because it indicates a proper level of awareness in this field; however, despite their favorable awareness, they do not have proper performance in this respect. Nevertheless, some important items such as transfer of *S. mutans* from the oral cavity of parents to the oral cavity of children were reported by a limited number of parents; therefore, knowledge in this aspect should be promoted in the community. Maybe the most important achievement of the present study was the attention of some parents to the role of their chronic stress in the occurrence of S-ECC, which is a new subject and further studies are necessary on it as a co-etiologic factor for S-ECC. Another important consideration was the fact that unfortunately none of the parents mentioned the important role of the quality and quantity of saliva in preventing S-ECC, which should be promoted in the community.

## Conflict of Interests

The authors declare no conflict(s) of interest related to the publication of this work.

## Authors’ Contributions

The concept and the design of the study were developed by HP. The interviews were done by MB. Data entry and statistical analyses were carried out by MB and MD and MS. The manuscript was written by AHA and HP. The proof reading was carried out by AHA and HP. All the authors participated in the literature review.

## Acknowledgments

This study was supported by grants from Kerman University of Medical Sciences. The authors would like to thank the staff at the Departments of Pediatric Dentistry for their assistance.

## Funding

Study was not funded by any institution or organization except Kerman University of Medical Sciences.

## Ethics Approval

The study was approved by the Institutional Ethical Committee at Kerman University of Medical Sciences, Iran with Ethical Code IR.KMU.REC.1394.464.
